# The Role and Mechanism of Gut Microbiota and Metabolites in Vascular Calcification

**DOI:** 10.3390/ijms27031364

**Published:** 2026-01-29

**Authors:** Xing-Yu Cao, Ao-Yuan Zhang, Ke-Feng Li, Yi-Wen Bie, Gui-Wen Xu, Chu-Yue Zhou, Xiao-Yue Ma, You-Yi Zhuang, Hai-Jian Sun, Xue-Xue Zhu

**Affiliations:** MOE Medical Basic Research Innovation Center for Gut Microbiota and Chronic Diseases, School of Medicine, Jiangnan University, Wuxi 214122, China; medic0311@163.com (X.-Y.C.); aoyuanzhang00@163.com (A.-Y.Z.); 1280124226@stu.jiangnan.eu.cn (K.-F.L.); bieyiwen@163.com (Y.-W.B.); 6242819002@stu.jiangnan.edu.cn (G.-W.X.); 1280124118@stu.jiangnan.edu.cn (C.-Y.Z.); 1282230212@stu.jiangnan.edu.cn (X.-Y.M.); 1282230430@stu.jiangnan.edu.cn (Y.-Y.Z.)

**Keywords:** vascular calcification, gut microbiota, metabolites, therapeutic strategies

## Abstract

Vascular calcification (VC) is a pathological process involving the deposition of mineral salts within the vascular wall, representing a significant risk factor for the development and progression of cardiovascular disease. The gut microbiota refers to the diverse microbial ecosystem inhabiting the gastrointestinal tract, including bacteria, fungi, viruses, and other microorganisms. This community exhibits considerable variability in both population density and taxonomic composition, with current estimates indicating approximately 10^13^–10^14^ microorganisms residing in the human gut. Recent studies suggest that metabolites produced by the gut microbiota may influence the pathogenesis of VC through the gut–vascular axis. This review consolidates current findings on the molecular mechanisms driving VC and examines the potential contribution of gut microbiota dysbiosis to vascular pathology. Particular attention is given to the functional roles of microbial metabolites such as short-chain fatty acids (SCFAs), trimethylamine N-oxide (TMAO), lipopolysaccharide (LPS), uremic toxins, secondary bile acids, and vitamin K in modulating calcific processes. In addition, current limitations in the existing literature are outlined, and potential therapeutic approaches, including probiotic use, prebiotic interventions, and targeted dietary strategies, are discussed in the context of their relevance for future clinical management of VC.

## 1. Introduction 

Vascular calcification (VC), defined by the pathological accumulation of calcium ions and hydroxyapatite within the arterial wall [1], is closely associated with aging and smoking. It frequently accompanies cardiovascular disorders, including atherosclerosis, hypertension, diabetes mellitus, and chronic kidney disease (CKD)-associated vasculopathy [2,3]. Both thoracic aortic calcification and coronary artery calcification exhibit age-dependent prevalence patterns, posing significant health concerns for middle-aged and older populations [4]. Despite its clinical relevance, current medical practice lacks effective therapeutic strategies capable of preventing or reversing VC, partly due to the unavailability of definitive diagnostic biomarkers [5]. As such, elucidating the underlying mechanisms and identifying viable treatment approaches remain critical priorities.

The human microbiome, often regarded as a “second genome,” significantly influences host physiological processes. It encompasses diverse microbial taxa, including *Actinobacteria*, *Bacteroidetes*, *Firmicutes*, *Clostridium*, and *Proteus*, which inhabit various body sites such as the gastrointestinal tract, skin, oral cavity, and nasal passages [6]. Increasing evidence indicates that the gut microbiota plays an important role in modulating health and disease states, including vascular conditions [7]. VC is no exception. Recent findings suggest that gut microbiota and its metabolic products contribute to the development and progression of VC. However, most existing research has emphasized alterations in microbial composition among individuals with VC or examined the effects of individual gut-derived metabolites on calcification. A comprehensive account of the mechanisms by which gut microbiota influences VC remains lacking. This review aims to summarize how gut microbiota and key metabolites affect VC, assess evidence strength, and suggest research and clinical priorities.

## 2. Vascular Calcification 

### 2.1. Classification and Cellular Mechanisms Involved in VC

Arterial calcification is generally categorized into two forms: medial calcification and intimal calcification. Medial calcification, commonly associated with CKD, is characterized by widespread mineral deposition along the elastic lamina of the arterial media, resulting in reduced vascular elasticity [8]. This pathological stiffening contributes to decreased diastolic blood pressure, increased systolic blood pressure and pulse pressure, and elevated cardiac afterload, thereby exacerbating cardiovascular complications [9]. In contrast, intimal calcification frequently occurs in advanced stages of type 2 diabetes mellitus (T2DM) [10]. As a shared pathophysiological mechanism in cardiovascular and metabolic diseases, arterial calcification in these contexts is closely linked to metabolic disturbances, including dysregulation of glucose and lipid metabolism, alongside inflammatory responses and impaired autophagy [11]. 

Among the key cellular contributors to VC are vascular smooth muscle cells (VSMCs), which undergo pathological transformation under mineralizing conditions. This includes endothelial-to-mesenchymal transition and phenotypic switching toward an osteoblast-like profile, thereby promoting calcific deposition [12]. These cells upregulate transcription factors involved in osteogenesis and chondrogenesis, facilitating extracellular matrix mineralization. Concurrently, contractile protein expression is diminished, accelerating the calcification process [13]. 

Calcified VSMCs exhibit elevated expression of pro-calcific mediators such as bone morphogenetic protein 2 (BMP-2), Runt-related transcription factor 2 (Runx2), alkaline phosphatase (ALP), osteopontin, osteocalcin (OCN), receptor activator of nuclear factor kappa-Β ligand, and Msh homeobox 2 (MSX2) [14]. This is accompanied by a marked reduction in smooth muscle markers, including α-smooth muscle actin (α-SMA) and SM22α [15]. Additionally, the calcification process is modulated by inhibitors such as pyrophosphate and matrix Gla protein (MGP) [16]. 

BMP-2, a member of the TGF-β superfamily, is the most significant regulatory factor involved in the physiological ossification and repair of bones, osteogenic differentiation and osteoblast proliferation [17]. Runx2, formerly designated as Cbfa1/Pebp2αA/AML3, is essential for osteoblast lineage commitment [18], It also exerts transcriptional control over targets such as X-C motif chemokine receptor 1, further supporting osteogenic differentiation [19]. BMP-2 transmits its signal via BMPR-I, activating Smad1 and Smad5 as intracellular effectors [20,21]. This signaling cascade inhibits VSMC phenotypic conversion, thereby mitigating VC progression [22]. Studies of aortic valve calcification have shown that Smad1/5 activation may also involve the bidirectional regulation of p38 signaling [23]. Conversely, other findings suggest that p38 and JNK pathways may promote the expression of osteogenic genes such as Runx2 [22]. Runx2 functions as a key downstream effector of the BMP pathway, with Smad proteins directly interacting with the Runx2 transcriptional complex [24]. 

VC is closely associated with chronic, nonspecific inflammation. Interleukin (IL)-6 has been shown to regulate the BMP-2–Wnt/β-catenin signaling cascade, thereby promoting VC [25]. This regulation may be related to the subsequent activation of Runx2 [26]. A study has shown that β-catenin increases the mRNA and protein levels of Runx2 [27]. TNF-α, primarily secreted by macrophages, directly induces MSX2 expression via the NF-κB pathway, which subsequently increases the expression of ALP—a key enzyme involved in mineralization in VSMCs—thus contributing to VC [28,29]. In addition, NF-κB signaling plays a central role in the upregulation of calcifying mediators, including BMP-2 and Runx2 [30]. IL-18 promotes calcification and osteogenic differentiation of VSMCs through activation of the transient receptor potential melastatin 7 channel [31]. IL-1β acts as a major driver of calcium deposition in VSMCs by initiating osteogenic transcriptional programs. Elevated IL-1β expression has been observed in calcified coronary arteries of patients [32]. Interestingly, activation of Smad2/3 and Smad4 has been reported to inhibit the production of pro-inflammatory cytokines such as TNF-α while enhancing the expression of anti-inflammatory cytokines such as IL-10 [33]. 

The Notch signaling pathway, comprising a family of highly conserved cell surface receptors encoded by the Notch gene, regulates several processes in normal cell morphogenesis, including differentiation of pluripotent progenitor cells, apoptosis, and proliferation [34]. Studies have shown that lipoprotein(a) [Lp(a)] induces VC through both the Notch1–NF-κB and Notch1–BMP2–Smad1/5/9 pathways. NF-κB silencing attenuates Lp(a)-induced VC [35]. Moreover, miR-34a promotes aortic calcification by upregulating Runx2 via Notch1 [36], indicating that the Notch signaling pathway may serve as a regulatory target in VC.

Reactive oxygen species (ROS) are partially reduced oxygen derivatives generated in biological systems. These include oxygen radicals such as superoxide anion (O_2_·^−^) and hydroxyl radicals (·OH), as well as non-radical oxidants such as hydrogen peroxide (H_2_O_2_) and singlet oxygen (^1^O_2_). Excessive oxidative or reductive stress resulting from ROS imbalance can cause severe cellular injury and inappropriate cell death, potentially leading to organ dysfunction or systemic failure [37]. Mitochondrial dysfunction contributes to elevated ROS production and inflammation, which may induce osteogenic transdifferentiation and apoptosis of VSMCs, thereby promoting VC [38]. Additional studies suggest that ROS activate Runx2 via the PI3K/AKT/mTOR signaling pathway, further contributing to vascular mineralization [14].

Inflammatory responses associated with TGF-β1 signaling also influence calcification, particularly in CKD models and cultured human aortic smooth muscle cells (HASMCs) maintained in osteogenic medium. Upon TGF-β1 binding to its receptor TGFBR1, Smad2/3 becomes phosphorylated, facilitating its association with Smad4. This complex plays a central role in downstream signal transduction. Downregulation of Smad4 has been linked to the development of inflammation-related vascular pathology [33].

Ferroptosis represents a distinct form of regulated cell death, separate from apoptosis, necrosis, and autophagy [39]. It is primarily characterized by extensive lipid peroxidation of cellular membranes, which results in toxic ROS accumulation [40,41]. Seto et al. reported that iron supplementation attenuates VC by suppressing the osteogenic transdifferentiation of phosphate transporters and VSMCs [42]. In CKD contexts, inhibition of the SLC7A11/GSH/GPX4 axis promotes ferroptosis in VSMCs, thereby facilitating VC [43]. Moreover, suppression of p53 in VSMCs leads to reduced SLC7A11 expression, diminished glutathione biosynthesis, increased ROS deposition, and ferroptotic cell death, all contributing to calcification [44]. 

Collectively, these findings indicate that inflammatory mediators, such as IL-1β, NF-κB, IL-6, and TNF-α, as well as signaling cascades involving ROS and ferroptosis, are integral to the pathogenesis of VC. The underlying mechanisms are multifactorial and highly interconnected. 

### 2.2. Intracranial Artery Calcification, Carotid Artery Calcification and Vertebral Artery Calcification

Intracranial artery calcification (IAC), a hallmark radiographic feature of intracranial arteriosclerosis on CT scans, is an established risk factor for stroke [45] and dementia [46]. Current studies have shown that there are three patterns of intracranial arterial calcification, including intimal calcification, medial calcifications (characterized by mineralization of the internal elastic lamina) [47], and adventitial calcification. These calcification types demonstrate differential clinical manifestations. Intimal calcification is an atherosclerotic lesion, usually related to arterial stenosis. In contrast, autopsy findings reveal that medial calcification in the intracranial internal carotid artery (iICA) exhibits histologically non-atherosclerotic characteristics, associated with arterial stiffening, diminished arterial compliance and limited distensibility [48]. This can result in an increase in pulse wave velocity and pulse pressure, subsequently leading to chronic damage to brain tissues [49]. This distinct pathophysiology underlies both the slower progression of cerebral artery calcification compared to coronary counterparts and a temporal dissociation spanning approximately two decades between the peak incidence of cardiovascular diseases (such as coronary heart disease and myocardial infarction) and cerebrovascular diseases (often stroke).

Asymptomatic mineralization of carotid and vertebral arteries and their intracranial continuations is a common incidental discovery on CT imaging, reflecting distinct pathophysiological processes from coronary calcification [50]. In pediatric period, internal carotid artery (ICA) calcification predominantly localizes to the supraclinoid segment and carotid siphon as non-atherosclerotic internal elastic lamina deposits, while vertebral artery calcification preferentially involves the V4 segment [51]. In adults, both ICA siphon and vertebral artery calcification volumes increase with age, with the highest prevalence in Asian populations [52].

### 2.3. Related Manifestations and Specific Molecular Mechanisms of VC

**Figure 1:**
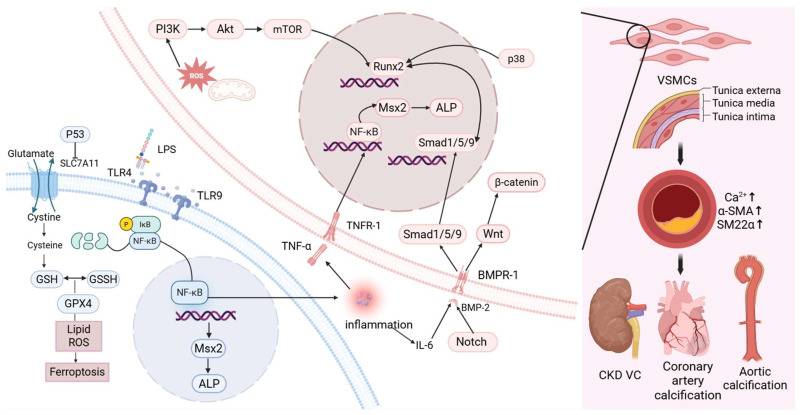
The related manifestations of VC and the corresponding cellular and molecular pathways. (Created in BioRender. c, X. (2026) https://BioRender.com/yae1im8).

From a macroscopic perspective, the development of VC is complex, with VSMCs undergoing osteogenic differentiation under conditions of mineral imbalance and various external stimuli. At the molecular level, the NF-κB signaling pathway plays a central role in VC pathogenesis. It facilitates the activation of several downstream calcification pathways, most notably the BMP2–Smad1/5–Runx2 axis and the NF-κB–Msx2–ALP cascade, by stimulating the release of pro-inflammatory cytokines. Additionally, ferroptosis-related mechanisms and ROS-induced cellular injury also contribute to the progression of VC. While detailed exploration of these molecular interactions falls beyond the scope of this review, the present work focuses on assessing whether gut microbiota-derived metabolites can modulate VC through these or related mechanisms (Figure 1).

## 3. The Changes in the Intestinal Flora of Patients with Various Types of Vascular Calcification

VC is recognized as a major contributor to cardiovascular morbidity and mortality [53]. It is highly prevalent in patients with CKD and is frequently observed as a complication in those with end-stage renal disease [54,55]. Studies have demonstrated that the gut microbiota of individuals with VC, particularly VC secondary to CKD, differs markedly from that of healthy controls. Hao et al. reported a significant increase in the abundance of *Prevotella copri* in CKD rats with aortic calcification. Administration of *P. copri* to the aortas of CKD rats led to reduced expression of the contractile marker α-SMA, alongside increased expression of osteogenic markers Runx2 and BMP2 [56], suggesting its involvement in promoting VC. 

In advanced CKD, dialysis is routinely employed to maintain physiological stability. Bao et al. examined the relationship between gut microbiota and VC in hemodialysis (HD) patients using abdominal aortic calcification (AAC) scores. Their findings indicated that patients with higher AAC scores exhibited significantly reduced overall fecal microbial diversity. In this group, *Proteobacteria* were more abundant, while *Bacteroidetes* and *Firmicutes* were notably less represented. At the genus level, *Escherichia-Shigella*, *Ruminococcus gnavus* group, and *Lactobacillus* were enriched in patients with high AAC scores. Conversely, *Ruminococcus* and *Lachnospiraceae_NK4A136_group* were more prevalent in individuals with low AAC scores. Among these taxa, *Escherichia-Shigella* had the greatest influence on VC progression in HD patients [57]. Merino-Ribas et al. conducted a study on CKD patients undergoing peritoneal dialysis (PD) and observed distinct microbial signatures associated with VC. In patients with VC, there was an increased abundance of *Cutibacterium*, *Pajaroellobacter*, *Devosia*, and *Hyphomicrobium*, while *Pelomonas* was relatively depleted. Changes were also noted in the abundance of *Coprobacter*, *Coprococcus 3*, *Lactobacillus*, and *Eubacterium eligens* group [58].

Yu et al. reported that *Adlercreutzia* and *Alistipes* were negatively correlated with VC, with the increased abundance of *Adlercreutzia* potentially linked to enhanced production of SCFAs [59]. Liu et al. examined differences in the gut microbiome of patients with high and low aortic arch calcification (AoAC) scores [60], noting distinct microbial community structures among those with lower scores. Specifically, greater abundance was observed for *Agathobacter*, *Eubacterium coprostanoligenes* group, *Ruminococcaceae UCG-002*, *Barnesiella*, *Butyricimonas*, *Oscillibacter*, *Ruminococcaceae DTU089*, and *Oxalobacter*. By contrast, the relative abundance of *Bacilli* was elevated in the high AoAC group [61]. 

In a study of Japanese men, Okami et al. investigated gut microbiota and CAC. They found that the Firmicutes-to-Bacteroidetes ratio, as well as the relative abundance of *Lactobacillus*, positively correlated with CAC scores and the presence of CAD. Furthermore, a positive association was identified between *Enterobacteriaceae* and CAC, while the genus *Blautia* appeared protective against CAD but showed no significant association with CAC progression [62]. Sun et al. further observed that increased abundance of *Acinetobacter* species in the bloodstream correlated positively with calcification of the kidneys and thoracic aorta, promoting Ca^2+^ precipitation in VSMCs [63]. Similarly, Yan et al. demonstrated, through metabolomic analysis, that compared to controls, patients with VC exhibited reduced Firmicutes-to-Bacteroidetes ratios, alongside decreased abundance of *Firmicutes*, *Muribaculaceae*, and *Alloprevotella*. Conversely, *Bacteroidetes*, *Proteobacteria*, *Bifidobacterium*, and *Escherichia-Shigella* were significantly enriched in the VC group [64]. The summary of gut microbiota changes associated with VC is shown in Table 1.

Collectively, these studies demonstrate a correlation between microbial alterations and VC. However, whether such changes represent causal factors or secondary phenomena remains unresolved. Moreover, microbial patterns associated with VC vary across species and treatment conditions. These inconsistencies may reflect the inherent diversity of microbial communities, differences in experimental approaches, or species-specific responses. Larger cohort-based investigations are required to clarify the causal relationship between gut microbiota and VC.

## 4. The Role of Gut Microbiota and Its Metabolites in the Development of Vascular Calcification

### 4.1. Short-Chain Fatty Acids

Fatty acids are generally classified into short-chain, medium-chain, and long-chain forms based on carbon chain length. Among these, acetic acid, propionic acid, and butyric acid are common Short-Chain Fatty Acids (SCFAs). They are the end products of dietary fiber fermentation by gut microbiota under anaerobic conditions and are typically present at high concentrations in the proximal colon [65,66]. A wide range of gut microbes participate in SCFA production, with members of the *Bacteroidetes* and *Firmicutes* phyla serving as primary contributors [67]. For example, *Bifidobacterium* species produce acetic acid, formic acid, lactic acid, and succinic acid; *Blautia* and *Lactobacillus* species generate acetic and lactic acid; and butyrate can be derived from *Anaerostipes*, *Eubacterium*, *Anaerostipes caccae*, and *Roseburia intestinalis* [68]. Propionate is primarily synthesized by *Bacteroidetes* via the succinate pathway, while butyrate production in *Firmicutes* occurs mainly through the acetyl-CoA pathway. *Ruminococcus*, a Gram-positive anaerobe belonging to the *Firmicutes* phylum, ferments polysaccharides to yield SCFAs [69,70,71]. The *Bacteroidetes* phylum also contributes to SCFA biosynthesis through metabolic transformations involving the tricarboxylic acid cycle, pyruvate metabolism, and starch and sucrose utilization. Consequently, reduced *Bacteroidetes* abundance may impair SCFA production [72,73]. Other SCFA-producing taxa include *Butyricimonas* [74] and *Bani* [75] are also intestinal microorganisms that produce SCFAs.

SCFAs contribute to intestinal barrier integrity, regulate glucose and lipid metabolism, modulate immune and inflammatory responses, and influence blood pressure homeostasis. Increased SCFA availability is therefore considered beneficial for cardiovascular and metabolic health [76]. Yan et al. reported that *Muribaculaceae* and *Akkermansia* showed positive correlations with SCFA levels in plasma and feces, whereas *Escherichia-Shigella*, *Bilophila*, and *Parasutterella* exhibited negative correlations. Propionate has been shown to attenuate VC in rats with vitamin D_3_ plus nicotine -induced disease by reducing inflammatory activity and protecting the intestinal mucosa through enhanced SCFAs generation. In this context, *Akkermansia* played a key role in fostering beneficial microbial populations that promote SCFA synthesis [64]. Moreover, acetic acid, propionic acid, and butyric acid can activate host receptors, suppress pro-inflammatory mediators such as TNF-α, IL-4, IL-5, IL-17a, and IL-6, and thereby mitigate VC [64,77].

Studies have confirmed that acetic acid, propionic acid, and butyric acid mediate signal transduction through surface-expressed free fatty acid receptors or via G protein-coupled receptors (GPCRs) such as GPR41, GPR43, and GPR109A, subsequently activating downstream signaling cascades. Both GPR41 and GPR43 respond to acetate, propionate, and butyrate, whereas GPR109A is primarily activated by butyrate [68]. Current research on SCFAs has largely emphasized their capacity to regulate inflammatory processes, which may underlie their protective role against VC. Zeng et al. reported that antibiotic treatment can enhance VC by reducing serum SCFA concentrations. They further demonstrated that acetate derived from gut microbiota attenuates VC [78], likely through inhibition of VSMCs osteogenic transformation. This mechanism may involve GPR43 signaling, which suppresses the NF-κB pathway and decreases the release of TNF-α and IL-6 [79]. In intestinal epithelial cells, GPR43 can also activate the NLRP3 inflammasome and increase IL-18 production [80]. Whether VSMCs employ a comparable mechanism remains to be established.

SCFAs additionally interact with GPR109A, modulating macrophage activity and suppressing inflammatory responses [67]. Butyric acid and propionic acid inhibit histone deacetylase (HDAC) activity, thereby exerting anti-inflammatory effects on intestinal epithelial cells, macrophages, and dendritic cells [66]. Their influence on bone metabolism may be mediated through regulatory T cells (Tregs), which are known to suppress osteoclastogenesis and enhance bone mass [81]. Regulation of Tregs by SCFAs may therefore represent another pathway through which these metabolites influence VC development. Notably, SCFAs may also exert pro-inflammatory effects under certain conditions. Studies indicate that when activated via Toll-like receptors (TLRs), SCFAs can regulate inflammation by inhibiting HDAC activity, reducing IL-10 expression, and downregulating the cell-type Fas-associated death domain-like IL-1β converting enzyme inhibitor protein. These changes promote NLRP3 inflammasome activation and IL-1β release, thereby intensifying inflammatory responses [82] and potentially accelerating VC. 

### 4.2. Lipopolysaccharide

Lipopolysaccharide (LPS), the major pro-inflammatory component of the Gram-negative bacterial cell envelope, is recognized by TLRs, thereby initiating downstream signaling that promotes pro-inflammatory cytokine synthesis [83]. This inflammatory response may be further amplified in the context of VC. For example, LPS derived from *P. copri* binds to TLR4 and markedly induces osteogenic differentiation and calcification of VSMCs by activating the NF-κB pathway, concomitant with increased expression of TNF-α, IL-1β, and IL-6 [56]. 

Su et al. reported that oxidized low-density lipoprotein enhances BMP-2 expression in human coronary artery endothelial cells through both TLR2 and TLR4. This effect involves ERK1/2 and NF-κB signaling cascades [84]. Similarly, Zhao et al. demonstrated that LPS activates TLR4 and TLR9, promoting calcification and osteoblast differentiation in HASMCs. Inhibition of the TLR9/NF-κB/BMP-2 axis was shown to attenuate VC associated with chronic renal failure [85], These findings suggest that NF-κB-mediated regulation of BMP-2 expression may occur through pro-inflammatory mediators such as IL-6 and TNF-α. Notably, TNF-α itself is a potent stimulator of vascular cell mineralization [86]. In conditioned medium, LPS activates monocytes and macrophages through TNF-α, which in turn enhances ALP activity and osteogenic differentiation of calcifying vascular cells [87]. 

Taken together, these observations indicate that LPS contributes to VC by activating TLR signaling, engaging the NF-κB pathway, and inducing pro-inflammatory cytokine production. Additionally, LPS promotes TNF-α-mediated upregulation of ALP, thereby intensifying VC.

**Figure 2:**
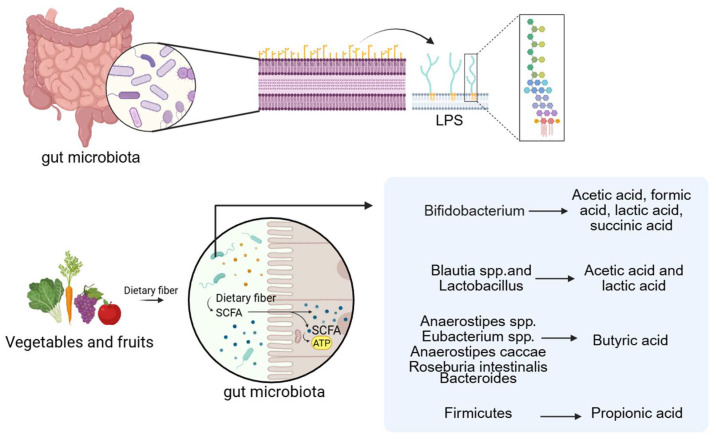
LPS and SCFAs are the related metabolites of the gut microbiota. (Created in BioRender. c, X. (2026) https://BioRender.com/wqxcil4).

Of further significance, the macrophage murine cell line RAW 264.7 treated with lipopolysaccharide (LPS-EK) derived from *Proteobacteria* species such as *Escherichia coli*, *Vibrio cholerae*, and *Helicobacter pylori* reduces macrophage-derived extracellular vesicles (EVs). EVs exposed to LPS-EK are enriched in pro-inflammatory cytokines as well as CAD-related proteins, including plasminogen activator inhibitor-1 (PAI-1) and serum amyloid A-3 protein (SAA3). These vesicles activate TLR4, with SAA3 specifically inducing pro-inflammatory cytokine release and osteogenic differentiation of mesenchymal stem cells via TLR4 signaling [88]. This mechanism may represent another pathway by which LPS contributes to VC (Figure 2).

### 4.3. Uremic Toxin from End-Stage Renal Disease

CKD is associated with intestinal dysbiosis and impaired nutrient absorption, leading to increased production of uremic toxins derived from microbial metabolism. These include trimethylamine N-oxide (TMAO), p-cresol sulfate (PCS), indoxyl sulfate (IS), and indole-3-acetic acid (3-IAA). Such toxins elevate oxidative stress and sustain chronic systemic inflammation, thereby amplifying the risk of CKD-related cardiovascular disease (CVD) [89,90]. In CKD rat models, *Eggerthella lenta* and *Fusobacterium nucleatum* enhance the generation of uremic toxins and accelerate disease progression [91]. IS and PCS, both products of amino acid metabolism, involve intestinal *Escherichia coli* and *Shigella*, indicating that increased abundance of *Escherichia-Shigella* is linked to VC [57]. Among these toxins, IS plays a particularly important role in promoting VC. Barreto et al. demonstrated a positive correlation between serum IS, PCS, and VC in CKD patients [92]. Similarly, Opdebeeck et al. reported that chronic exposure of CKD rats to IS and PCS activated inflammatory and coagulation pathways, thereby accelerating VC progression [93]. 

The aryl hydrocarbon receptor (AhR), abundantly expressed in vascular tissues, is critically involved in vascular pathology [94]. During interactions between gut microbiota and host cells, tryptophan metabolites and SCFAs are generated, both of which can activate AhR [95]. AhR, an intracellular receptor originally identified for dioxins and dioxin-like compounds, also serves as a receptor for IS [96]. Experimental studies have shown that AhR agonists such as IS and hexachlorobenzene can induce hypertension and vascular dysfunction in mice [97,98]. IS-mediated AhR activation stimulates ROS production, contributing to oxidative stress, inflammation, and endothelial cell senescence [99,100,101]. This pathway also activates NF-κB in endothelial cells, upregulating vascular adhesion molecules such as ICAM-1 and MCP-1, thereby increasing susceptibility to VC and atherosclerosis [102]. 

In addition, IS reduces nitric oxide production while enhancing ROS generation, leading to progressive endothelial injury [103]. AhR dysregulation further promotes MAPK/NF-κB signaling, triggering inflammation without activating the NLRP3 inflammasome. This may explain the persistence of chronic, low-grade inflammation induced by IS in CKD patients [104]. Collectively, these findings suggest that gut microbiota-derived metabolites, including tryptophan derivatives and SCFAs, may activate AhR, drive ROS production, and trigger inflammatory pathways in vascular endothelial cells. Such processes likely contribute to the initiation and progression of VC.

IS has also been shown to regulate Wnt7b/β-catenin signaling via microRNA-29b in HASMCs, thereby reducing intracellular RUNX2 levels and attenuating VC [105]. Sun et al. further demonstrated that IS exposure activates the renal renin–angiotensin–aldosterone system (RAAS), with increased expression of renin, angiotensinogen, and AT1R proteins. Their work also revealed that IS stimulates RAAS activation through the TGF-β pathway [106]. This pathway may represent a mechanistic link between IS and VC, although further validation is required.

Another uremic toxin, PCS, promotes endothelial dysfunction by upregulating TNF-α, MCP-1, and ICAM-1, thereby contributing to atherosclerosis. This effect is mediated by the induction of NADPH oxidase activity, leading to ROS generation [107,108].

Emerging evidence also suggests that macrophage polarization may play a role in VC progression. Chen et al. reported that pravastatin enhances the activity of *Bacteroides* by promoting ArsR expression, which in turn increases production of 3,4,5-trimethoxycinnamic acid (TMCA). TMCA was shown to promote VC in type 2 diabetes by inducing macrophage polarization toward the pro-inflammatory M1 phenotype [109]. Conversely, *Bacteroides fragilis* has been implicated in vesicle-mediated activation of STING signaling, Trib1, and Mef2d genes, driving macrophage polarization toward the M2 phenotype and thereby facilitating VC [110]. IS has similarly been associated with skewing macrophages toward the M1 phenotype, while also possessing the capacity to induce M2 polarization. This dual modulatory effect may contribute to vascular remodeling in atherosclerotic lesions among CKD patients [111]. Whether IS actively promotes VC through macrophage polarization, however, remains to be clarified.

### 4.4. Trimethylamine N-oxide

Studies have shown that administration of metronidazole, which alters gut microbiota composition, leads to a marked reduction in serum TMAO levels [112], suggesting a strong association between TMAO production and microbial activity. In humans, compounds such as choline, L-carnitine, and betaine are metabolized by intestinal microbiota into trimethylamine (TMA), which is subsequently oxidized in the liver by flavin-containing monooxygenases (FMOs) into TMAO [113]. Multiple international cohort studies have demonstrated that elevated TMAO levels increase the risk of CVD and promote atherosclerosis [114,115], as well as raising the likelihood of heart failure [116,117].

In vitro experiments involving mouse bone marrow-derived macrophages revealed that TMAO enhances the secretion of pro-inflammatory cytokines IL-6 and IL-12p40 while suppressing the anti-inflammatory cytokine IL-10. It also upregulates several inflammation-related genes, including *Nr4a3*, *H-Q5*, *H2-Q6*, *Irf3*, *Irf5*, and *Acod1* [112]. Ma et al. demonstrated that berberine can attenuate atherosclerosis by reducing microbial TMA production from choline, acting through vitamin-like modulation of gut metabolism [118]. 

TMAO has also been shown to promote VC by activating the NLRP3 inflammasome, enhancing NF-κB signaling, and inducing osteogenic differentiation in VSMCs [119]. In both VSMCs and endothelial cells, TMAO activates MAPK signaling pathways, contributing to inflammatory gene expression and promoting leukocyte adhesion [120]. Some studies suggest that high-choline diets elevate serum TMAO levels and facilitate NF-κB activation through endoplasmic reticulum–mitochondrial stress, promoting osteogenic responses in human aortic valves. However, this mechanism has not yet been investigated in VSMCs [121]. TMAO has also been implicated in exacerbating renal damage by increasing Smad3 phosphorylation, a key regulator of TGF-β signaling [122]. 

**Figure 3:**
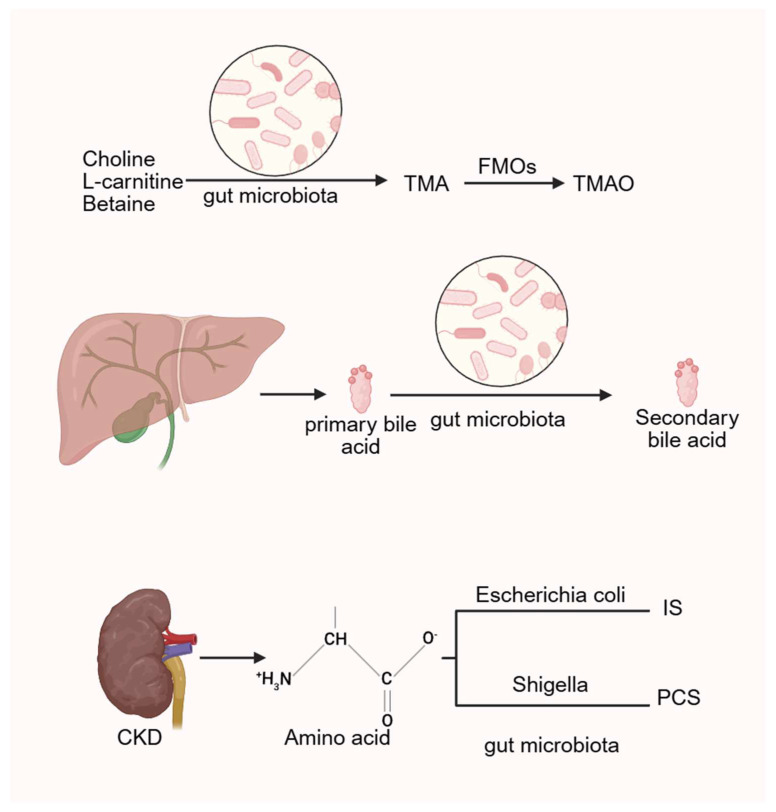
The gut microbiota generates TMAO, bile acids and related uremic toxins by regulating related metabolic processes. (Created in BioRender. c, X. (2026) https://BioRender.com/wz2634r).

Nevertheless, TMAO has been shown to increase calcium release from endoplasmic reticulum stores in platelets, promoting aggregation and thrombosis [123]. Overall, the role of TMAO in promoting atherosclerosis is well established, whereas its direct involvement in VC remains under investigation (Figure 3).

### 4.5. Other Related Mechanisms

Vitamin K2 (VK2) is synthesized through the metabolic activity of gut microorganisms, with approximately 50% of the body’s vitamin K originating from bacterial decomposition in the intestine. Key contributing genera include *Clostridium* and *Lactobacillus* [124]. These microbes also contribute to the maintenance of intestinal microbial homeostasis and possess coagulation-supporting properties that help prevent bleeding [125]. VK2 has been shown to modulate the gut microbiota composition, alleviate inflammation, and reduce oxidative stress in both the gastrointestinal and central nervous systems [126].

OCN, the most abundant non-collagenous protein in bone, plays an important role in both physiological and pathological calcification [127]. OCN can promote VC by activating hypoxia-inducible factor 1-alpha (HIF-1α), thereby enhancing mineralization in VSMCs, or by stimulating the Runx2 signaling pathway [128]. VK2 facilitates the carboxylation of OCN, thereby reducing aberrant calcium deposition in blood vessels. In addition, VK2 activates MGP, a known inhibitor of arterial wall calcification. Once phosphorylated, MGP acquires the capacity to bind calcium and hydroxyapatite, reducing vascular deposition of Ca^2+^ and mitigating VC risk [129]. 

*Oxalobacter formigenes* has been identified as a key organism in the intestinal degradation of oxalate and may lower the risk of calcium oxalate kidney stone formation [130]. Oxalate can induce reactive oxygen species, driving oxidative stress, inflammation, and damage to vascular endothelial cells [131]. A significant association between calcium oxalate stones and AAC has also been reported [132]. However, the precise mechanisms by which oxalate contributes to aortic calcification remain unclear and warrant further investigation. 

Bile acids are cholesterol-derived molecules primarily synthesized in the liver. Their signaling functions are mediated through various receptors, including the nuclear receptor farnesoid X receptor (FXR) and the membrane-bound Takeda G protein-coupled receptor 5, also known as the G protein-coupled bile acid receptor 1. In the gut, primary bile acids undergo microbial 7α-dehydroxylation to form secondary bile acids, with deoxycholic acid (DCA) being a principal product. This transformation is primarily facilitated by a limited number of anaerobic bacteria, such as *Clostridium*, via bile salt hydrolases (BSHs) [133].

DCA has been shown to activate the transferrin receptor–acyl-CoA synthetase long-chain family member 4 pathway, inducing ferroptosis [134]. In CKD, free DCA aggravates VC by inducing activating transcription factor 4 via endoplasmic reticulum stress. Conversely, FXR-specific agonists have been reported to reduce circulating bile acids, including DCA, in murine models, thereby preventing CKD-associated medial layer and atherosclerotic calcification [135]. Although elevated serum DCA levels are often observed in patients with advanced CKD and appear to be associated with CAC, cross-sectional analyses have not established a definitive correlation [136]. 

Lithocholic acid, another secondary bile acid produced by intestinal bacteria, serves as an agonist of the vitamin D receptor. Hashimoto et al. found that lithocholic acid enhances calcium and phosphate absorption in a vitamin D receptor–claudin-3 dependent manner. They also reported reduced serum levels of 1,25(OH)_2_ vitamin D in CKD patients [137]. These findings support the hypothesis that lithocholic acid may contribute to the development of VC, particularly in patients with impaired vitamin D metabolism. Therefore, the interplay between gut microbiota-derived bile acids and host signaling pathways likely plays a critical role in VC pathogenesis.

**Figure 4:**
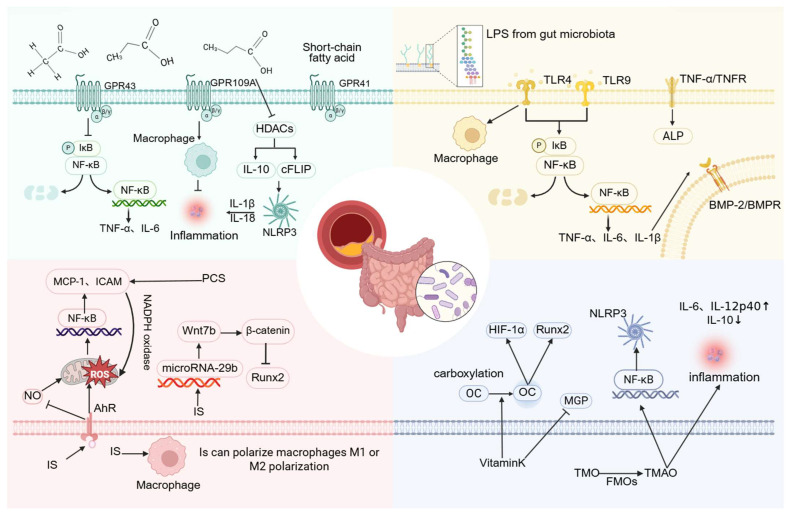
The molecular mechanisms by which various gut microbiota-related metabolites regulate vascular calcification. (Created in BioRender. c, X. (2026) https://BioRender.com/nap4i75).

Additionally, recent research suggests that multiple microbial species may modulate metabolite levels to influence VC progression. For instance, the microbial strain *gKLE1615* has been implicated in promoting VC by reducing the methionine-to-phosphate ratio. Key pathways involved include GDP-L-fucose biosynthesis, 5-aminoimidazole ribonucleotide biosynthesis, L-glutamine degradation, the S-adenosyl-L-methionine cycle, and thiazole biosynthesis [138] (Figure 4).

## 5. Methods and Measures for Preventing Vascular Calcification Through Modulation of Gut Microbiota

### 5.1. Dietary Modulation for VC Prevention

Dietary habits play an essential role in mitigating CVD risk. Experimental studies have shown that increased dietary fiber intake enhances SCFA production via microbial fermentation, while simultaneously increasing the abundance of *Firmicutes* and the relative proportion of *Proteobacteria*, particularly *Enterobacteriaceae*. In contrast, reduced fiber consumption leads to decreased production of indole-3-acetic acid from tryptophan, a change associated with diminished abundance of *Clostridium* species [139]. This reduction may lower AhR activation, potentially contributing to VC progression.

There is evidence suggesting that a high-choline diet promotes the microbial conversion of TMA, which is subsequently oxidized to TMAO in the liver. Conversely, a high-fiber diet facilitates the microbial conversion of cholesterol into bile acids, which then activates liver FMOs to enhance TMA oxidation and TMAO production [140]. Bile acids further stimulate FXR within the FMO gene family, promoting hepatic TMAO generation. Among dietary sources, TMAO derived from fish has the most substantial effect on circulating TMAO levels. Additionally, excessive caloric intake (>1000 kcal/day) and high-fat consumption (55% fat) over a period of four weeks have been shown to elevate fasting plasma TMAO concentrations [122]. 

Collectively, these findings suggest that high-choline diets may exacerbate VC by increasing gut-derived TMAO. In contrast, dietary fiber exhibits a dual effect: it may reduce VC through increased SCFA production but also contribute to TMAO elevation, depending on fiber type and intake patterns.

Interestingly, the choline analog 3,3-dimethylbutanol (DMB) has been shown to inhibit choline TMA lyase activity, thereby lowering systemic TMAO levels and attenuating choline-induced atherosclerosis. DMB is a naturally occurring compound found in certain foods such as balsamic vinegar, red wine, cold-pressed extra virgin olive oil, and grape seed oil [141].

### 5.2. Probiotics, Prebiotics, Synbiotics, and Aerobic Exercise in the Regulation of Vascular Calcification

Probiotic microorganisms influence a range of physiological processes. Their functions include pathogen defense, immune modulation, mineral absorption, and the regulation of metabolic activity [142]. Common probiotic strains belong to the genera *Lactobacillus*, *Bifidobacterium*, and *Streptococcus*, along with *Saccharomyces* [143]. *Lactobacillus* and *Bifidobacterium* have been shown to reduce levels of inflammatory mediators such as IL-1β in murine models of inflammatory bowel disease, contributing to the maintenance of intestinal barrier integrity [144,145]. Through suppression of systemic inflammation, these strains may help alleviate VC.

However, certain *Lactobacillus* strains have been reported to translocate across impaired intestinal barriers and colonize internal organs such as the liver, potentially inducing inflammatory responses [146] that could contribute to VC. In another study, researchers from China and Sweden identified *Eubacterium eligens* (*E. eligens*) as a promising microbial target for treating atherosclerosis and VC [147]. Based on this, transplantation of beneficial bacteria may represent a viable therapeutic strategy.

Innovative approaches, such as the development of bioinspired wrinkled microspheres loaded with probiotics (CSM@5), have been shown to significantly lower serum phosphorus levels while modulating gut microbiota composition. This intervention led to increased beneficial bacteria and decreased harmful species, ultimately improving VC outcomes [148]. 

Prebiotics, such as inulin, have been found to elevate SCFA levels and alter gut microbiota by increasing the abundance of specific bacterial genera, although without significant effects on plasma cholesterol or atherosclerosis progression [149]. In CKD models, synbiotic supplementation—including *Sialobacter* LBR228, *Bifidobacterium* BFS309, fructooligosaccharides, and chitosan oligosaccharides—has been effective in alleviating hyperphosphatemia and secondary hyperparathyroidism (SHPT), and in reducing VC induced by calcium deposition secondary to SHPT [150]. Resveratrol, a plant-derived antitoxin with prebiotic properties, reduces plasma TMAO levels in *ApoE*^−/−^ mice by reshaping the intestinal microbiota. This compound increases *Lactobacillus* and *Bifidobacterium* abundance while promoting bile acid synthesis [151]. 

**Figure 5:**
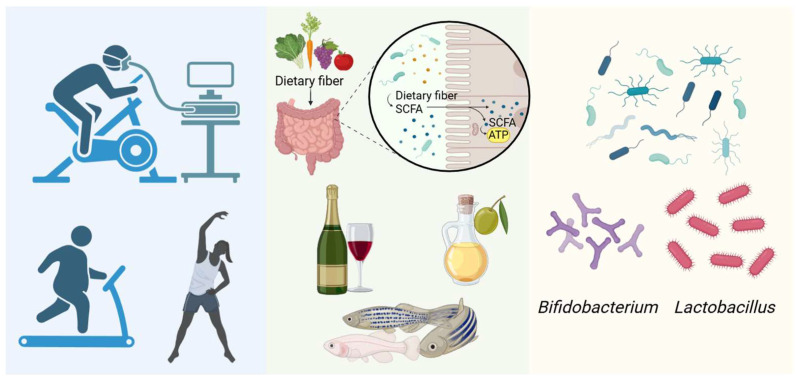
Relieving vascular calcification through the gut microbiota. (Created in BioRender. c, X. (2026) https://BioRender.com/kouu89w).

Physical activity may also influence VC through modulation of the gut microbiota. In athletes, the relative abundance of *Akkermansia* has been found to be significantly higher in those with lower body mass index, potentially promoting SCFA production and reducing VC risk. Long-term exercise has been associated with increased proportions of the *Firmicutes* phylum and *Ruminococcaceae* family, and decreased abundance of the *Lactobacillaceae* family, *Bacteroides* genus, and *Lactobacillus* genus [152]. These exercise-induced shifts in microbial composition may represent an additional pathway for VC prevention (Figure 5). The elderly elite athletes (aged 60–80) who mainly engaged in endurance training had significantly higher maximum oxygen uptake (VO_2max_) and total bone mineral deposition compared to their sedentary counterparts of the same age. A detailed analysis revealed a significant negative correlation between VO_2max_ and coronary artery calcification damage, and a significant positive correlation with the VC inhibitor fetuin-A, suggesting that long-term exercise can simultaneously inhibit coronary artery calcification and increase bone mineral content [153]. 

The elderly of the same age group who have lifelong exercise habits have better vascular elasticity compared to those without exercise habits. This indicates that establishing good exercise habits during youth has significant benefits for resisting VC in old age, maintaining vascular compliance and maintaining good vascular function [154].

## 6. Conclusions 

The gut microbiota and its metabolites play a central role in the initiation and progression of VC. Numerous studies have demonstrated that dysbiosis of the gut microbiota influences VC through a range of complex signaling pathways and physiological mechanisms. As described in this review, specific microbial taxa may modulate VC by participating in metabolic pathways such as the LPS/TLR/NF-κB axis, which promotes inflammatory responses; the Notch/BMP2/Smad signaling cascade, which mediates vascular mineralization; and the TNF-α pathway, which induces *Msx2* expression and subsequently enhances ALP activity, promoting calcification.

Key microbial metabolites, including IS, SCFAs, TMAO, and VK2, are involved in these regulatory mechanisms. These metabolites may activate signaling pathways that contribute to VC, although the precise nature of their interactions remains to be fully elucidated. In addition to dietary and probiotic interventions, other strategies such as fecal microbiota transplantation and antibiotic administration have also shown potential in modulating VC [64]. However, most evidence supporting these interventions is derived from animal models, and robust clinical validation is lacking.

Several studies have suggested that both gut microbiota and their metabolic products may influence aortic valve calcification. Although these processes share overlapping molecular mechanisms with VC, it is still unclear whether the effects observed in valve tissues are directly applicable to vascular wall calcification, particularly in the context of CKD or T2DM.

Despite considerable progress, many challenges remain. The complexity of gut microbial ecosystems and the dynamic gut environment limit our current understanding of the interactions between specific microbial species and their metabolites. Additionally, substantial inter-individual variability in gut microbiota composition and function complicates efforts to determine how these differences influence VC susceptibility and progression. Most existing research in this field is based on animal and in vitro models, and high-quality clinical data, especially randomized controlled trials, are still lacking.

Future research should aim to elucidate the molecular mechanisms through which gut microbiota and their metabolites regulate VC, integrating multi-pathway and multi-omics approaches. Such efforts could facilitate the development of novel therapeutic strategies targeting the gut microbiota for the prevention and treatment of VC.

## Figures and Tables

**Table 1:** Alterations in gut microbiota associated with vascular calcification.

Study Population/Model	Gut Microbiota Change	Direction of Change
**CKD rats with aortic calcification [56]**	Fecal bacterial species abundance	Decreased (in high AAC)
	Phylum Bacteroidetes	
	Phylum Synergistetes	
	Lachnospiraceae_NK4A136_group	
	Ruminococcus	
	Phylum Proteobacteria	Increased (in high AAC)
	Escherichia-Shigella	
	Ruminococcus gnavus group	
	Lactobacillus	
**PD-CKD [58]**	Cutibacterium	Increased (in VC group)
	Pajaroellobacter	
	Devosia	
	Hyphomicrobium	
	Pelomonas	Decreased (in VC group)
	Coprobacter	Changed (VC group)
	Coprococcus 3	
	Lactobacillus	
	E. eligens	
**Not Specified [59]**	Adlercreutzia	Increased (Negative correlation)
	Alistipes	
**Patients with chronic disease [61]**	Agathobacter	Increased (Low AoAC)
	Eubacterium coprostanoligenes	
	Ruminococcaceae UCG-002	
	Barnesiella	
	Butyricimonas	
	Oscillibacter	
	Ruminococcaceae DTU089	
	Oxalobacter	
	Bacilli	Increased (High AoAC)
**Japanese men [62]**	Firmicutes-to-Bacteroidetes Ratio	Increased (Positive correlation)
	Actinobacteria	
	Enterobacteriaceae	Correlation *
	Blautia genus	Decreased (Negative correlation †)
**CKD rats with thoracic aortic calcification [63]**	Acinetobacter (genus)	Increased (Positive correlation ‡)
**Mice with VC [64]**	Firmicutes/Bacteroidetes ratio.	Increased (VC group)
	Muribaculaceae	
	Alloprevotella	
	Bacteroidetes phylum	
	Proteobacteria phylum	
	Gemella genus	
	Escherichia_Shigella	

* Correlation: Refers to a statistically significant association between specific microbiota taxa and VC or related biomarkers, without implying causation. † Negative correlation: Denotes an inverse relationship where increased relative abundance of a microbial taxon correlates with reduced VC severity, as quantified by established calcification metrics. ‡ Positive correlation: Indicates a proportional relationship wherein elevated abundance of specific microbiota associates with higher VC burden, suggesting potential pathogenic involvement.

## Data Availability

No new data were generated or analyzed in this review. All data discussed are from published studies available through PubMed search.
